# Association of maternal central adiposity measured by ultrasound in early mid pregnancy with infant birth size

**DOI:** 10.1038/s41598-020-76741-8

**Published:** 2020-11-12

**Authors:** Emelie Lindberger, Anna-Karin Wikström, Eva Bergman, Karin Eurenius, Ajlana Mulic-Lutvica, Inger Sundström Poromaa, Fredrik Ahlsson

**Affiliations:** Department of Women’s and Children’s Health, Uppsala University, 751 85 Uppsala, Sweden

**Keywords:** Medical research, Epidemiology, Paediatric research

## Abstract

We sought to investigate whether early mid pregnancy visceral and subcutaneous fat depths measured by ultrasound were associated with infant birth size, independent of early pregnancy BMI. A cohort study was performed at Uppsala University Hospital, Sweden, between 2015–2018. Visceral and subcutaneous fat depths were measured at the early second-trimester anomaly scan in 2498 women, giving birth to singleton, term infants. Primary outcomes were birthweight and LGA (birthweight standard deviation score > 90th percentile in the cohort). Linear and logistic regression models were used, adjusted for BMI, age, smoking, parity, maternal country of birth, gestational age and infant sex. A 5-mm increase in visceral fat depth was associated with an increase in birthweight of 8.3 g [95% confidence interval (CI) 2.5 − 14.1 g], after adjustments, and a 6% increase in the adjusted odds of having an infant born LGA (OR 1.06, CI 1.02–1.11). There was no association between subcutaneous fat depth and birthweight or LGA after covariate adjustments. Hence, visceral fat depth measured by ultrasound in early mid pregnancy was associated with excessive fetal growth, independent of early pregnancy BMI, and may be useful in models for predicting LGA infants.

## Introduction

The epidemic of overweight and obesity continues worldwide. Almost 30% of women entering pregnancy globally are overweight, and about 10% have obesity^1^. In 2015–2016, 36% of U.S. women 20–39 years of age were obese^2^.

Overweight and obesity during pregnancy are risk factors for adverse pregnancy outcomes in both the mother and the infant^3–5^. Adverse neonatal outcomes include macrosomia, injuries to the nervous or skeletal system, respiratory distress syndrome, hypoglycemia, and seizures^6^. In addition, the infant has an increased risk of obesity when reaching adulthood^7^, creating a vicious inter-generational cycle of obesity^8^.

Central adiposity with excessive visceral fat accumulation is a stronger predictor of type 2 diabetes and cardiovascular disease, compared with general adiposity^9^. The precise mechanisms behind these findings are not fully understood, but central adiposity increases the risk of insulin resistance, hyperlipidemia, and low-grade inflammation^9,10^, which are proposed as the underlying causes of obesity-related conditions^11^.

Central adiposity during pregnancy is associated with macrosomic and large for gestational age (LGA) infants, independent of the mother’s body mass index (BMI)^12–18^. A macrosomic or LGA infant has a higher risk of complicated delivery^19^, fetal asphyxia^20^, shoulder dystocia^21^, plexus brachialis injury^22^, hypoglycemia^23^, and admission to neonatal intensive care unit^19,24,25^. Being born LGA may also have far-reaching consequences. Women born LGA have increased risk of developing overweight and obesity^26^, and are at increased risk of breast cancer as adults^27^. Both males and females born LGA have increased risk of type 2 diabetes mellitus and obesity^28^. Moreover, a mother born LGA has a doubled risk of giving birth to an LGA infant^26^. Of note, during the last decade, the proportion of infants born LGA has increased^29^.

Currently, BMI is used for risk stratification of pregnant women^30^, but the method does not differentiate central from peripheral adiposity, or adipose tissue from muscle tissue. It is suggested that central adiposity increases the risk of adverse pregnancy outcomes, and therefore could be a better marker of metabolic risk than BMI alone^12,31^.

Previous studies show associations between maternal central adiposity and infant birth size^12–18,32^, but the studies use several different methods to assess central adiposity^33^. To our knowledge, only two previous studies report on the association between ultrasound measurement of central adiposity including assessment of visceral fat tissue and infant birth size^15,32^, the first including only adolescent mothers^15^, and the second evaluating only 45 women^32^. Thus, larger studies performed with ultrasound measuring central adiposity differentiating visceral from subcutaneous fat tissue in clinical settings are lacking, addressing a gap within the existing literature. Therefore, the objective of this study was to investigate whether maternal visceral fat depth (VF) and subcutaneous fat depth (SCF) measured by ultrasound in early mid pregnancy were associated with infant birthweight and the likelihood of giving birth to an LGA infant, in a large population-based cohort.

## Material and methods

This was a cohort study performed between January 2015 and April 2018 at Uppsala University Hospital (Uppsala, Sweden). Ethical approval was obtained to implement a new clinical routine, ie the VF and SCF measurements, and to evaluate this routine by linkage to standardized hospital electronic medical records on maternal, obstetric, and perinatal health care. Following linkage, the study population database was anonymized. The study was approved by the Regional Ethical Review Board in Uppsala on September 24th 2014 (Dnr:2014/353), and performed in accordance with relevant national and international guidelines for medical research. Informed consent was waived by the Swedish Ethical Review Authority (Dnr: 2019-00391). As this was a register based study with anonymized data, informed consent was not required. In Sweden, large registry based studies generally do not require informed consent. Ludvigsson et al.^34^ explains that it is assumed that, as long as a registry-based study is deemed ethical by the ethical committee, the participants do not object to the research. The authors describe that this “assumed agreement to contribute personal data to research is part of the informal contract between the individual and the state (…), given that health care is traditionally virtually free of charge (…), and registry-based data are maintained for the purpose of health care quality improvement”^34^.

Eligible participants were women undergoing a second-trimester anomaly scan at 16–19 weeks of gestation at this hospital from January 2015 to December 2017 (n = 12,744), who had their scan performed by a midwife trained in VF and SCF measurements (n = 3027). It was a coincident which pregnant woman who was undergoing a scan that included fat depth measurements, since the personnel booking the second-trimester anomaly scan appointments was not involved in this study. Subsequently, 529 women were excluded from the final analyses: 163 were not possible to follow up, 52 had no available data on BMI at first antenatal visit, 7 had a multiple pregnancy, 1 had a miscarriage, 2 had intrauterine fetal death, 27 had missing birthweight data, and 267 had pre- or post term deliveries. In addition, 10 women were excluded because of missing VF or SCF measures. Hence, the study population consisted of 2498 women who gave birth to singleton, term infants between May 2015 and April 2018.

Additionally, we performed a sensitivity analysis where we restricted the population to healthy pregnancies. We excluded 49 women due to chronic illness (diabetes mellitus, rheumatic disease, epilepsy, inflammatory disease, or essential hypertension), 30 due to gestational diabetes mellitus, 75 due to gestational hypertension, and 83 due to preeclampsia. Hence, the healthy subgroup consisted of 2261 women and child dyads.

All second-trimester anomaly scans in Uppsala County are performed at Uppsala University Hospital, with scans performed in more than 97% of all pregnant women^35^. Hence, the study population was a population-based cohort. Moreover, Uppsala University Hospital is the only available delivery unit within the county, leading to excellent follow-up of participants.

### Data collection

Maternal VF and SCF were measured by ultrasound as per Armellini et al.^36^. The VF and SCF measurements were taken at the midline of the body with the measuring point 10 cm above the umbilicus. Visceral fat depth, measured in millimeters, was defined as the distance from the inner border of the rectus abdominis muscle to the anterior border of the aorta. Subcutaneous fat depth was defined as the distance from dermis to the surface of the rectus abdominis muscle, and measured in millimeters. Assessments were performed using a GE Voluson E6, E8 or E10 ultrasound machine (GE Medical Systems, Zipf, Austria). All participating midwives were certified obstetric ultra-sonographers. Additional training sessions were carried out during the study period to maximize the quality of the scans. The intraclass correlation coefficient of the inter-examiner variation in VF measurements was 0.83, and in SCF measurements the intraclass correlation coefficient was 0.85, both indicating good reliability^37^.

Maternal characteristics such as BMI (kg/m^2^), age (years), smoking status at first antenatal visit (yes or no), parity (nulliparous vs parous), and maternal country of birth (EU or outside EU) were obtained from the standardized antenatal electronic medical records. Early pregnancy BMI was categorized into the BMI classes defined by the World Health Organization: < 18.5 (underweight), 18.5–24.9 (normal weight), 25.0–29.9 (overweight), 30.0–34.9 (obesity class I), 35.0–39.9 (obesity class II), and ≥ 40 (obesity class III)^38^. We extracted information on chronic illnesses from checkboxes filled out by the midwife in the standardized antenatal electronic medical record at the first antenatal visit, and completed this information with diagnoses given by the obstetrician at discharge from the delivery unit. Information on the following diagnoses according to the International Classification of Diseases 10 (ICD-10) was obtained: diabetes mellitus (checkbox and E10, E11), rheumatic disease (check box and L40, M05, M32, M35, M45), epilepsy (check box and G40), inflammatory disease (check box and D69, K50, K51, K90), essential hypertension (checkbox and I10), gestational diabetes (O244), gestational hypertension (O13), and preeclampsia (O14). Gestational diabetes mellitus was defined as fasting plasma glucose ≥ 7.0 mmol/l or plasma glucose ≥ 9.0 mmol/l 2 h after oral intake of 75 g glucose. Term birth was defined as delivery after 37^+0^ to 41^+6^ weeks of gestation.

Information on infant birthweight, gestational age, and sex was extracted from the standardized pediatric electronic medical records. Birthweight standard deviation score (BWSDS) was calculated based on birthweight using the Swedish standards for sex and gestational age^39^. Small for gestational age was defined as BWSDS below the 10th percentile in the study cohort, and LGA as a BWSDS above the 90th percentile in the cohort.

### Statistical analyses

Normal distribution of the VF and SCF measures in the cohort was assessed. Differences in maternal VF and SCF between the World Health Organization BMI classes were determined by Kruskal–Wallis test followed by post-hoc paired tests with Bonferroni correction for multiple testing, since the standard deviations of VF and SCF were wide in the higher BMI classes. Pearson correlation coefficients were used to examine the associations between BWSDS, early pregnancy BMI, age, VF, and SCF.

Univariate linear regression analyses were used to separately examine the association between VF, SCF, and the ratio VF:SCF with birthweight and BWSDS. In addition, multivariable regression was conducted. In the models evaluating VF, adjustments were made for early pregnancy BMI, age, smoking at first antenatal visit, parity, SCF, country of birth, gestational age, and infant sex. In the models evaluating SCF, the same adjustments were made except VF was added instead of SCF. In the models evaluating the ratio VF:SCF, adjustments were made for early pregnancy BMI, age, smoking at first antenatal visit, parity, country of birth, gestational age and infant sex. Since maternal BMI is associated with increased birthweight^40^, BMI was adjusted for. Besides, maternal age is negatively associated with birthweight^41^, smoking is a risk factor of low birthweight^42^, and birthweight is increasing with parity^43^, motivating adjustment for these covariates. Lastly, birthweight increases with gestational age and boys are generally heavier at birth compared with girls^44^. The linear regression models were subsequently performed on the subgroup of healthy women, as described above.

Unadjusted logistic regression analyses were performed to separately examine the association between SCF, VF, and the ratio VF:SCF with the likelihood of giving birth to an LGA infant. Multivariable models were subsequently run, as described above. Adjustments were not made for infant gestational age and sex, since these variables already are included in BWSDS. All logistic regression models were also performed on the subgroup of healthy women.

Information on smoking at first antenatal visit was missing in 51% of the women. In order not to lose power in the multivariable regression analyses, we imputed data on smoking by random hot deck method^45^. Matched controls for all women with known smoking status were identified among women with missing information on smoking using information on age, BMI, and country of birth. Then, a random control was drawn for every individual with known smoking status. This random control was given the same smoking status as its matched individual with known smoking status. The prevalence of smoking in the age group 16–44 years in Sweden is 5%^46^. Since the majority of smoking women in Scandinavia stop using tobacco when they become pregnant^47^, the true prevalence is probably lower. After imputation, the prevalence of smoking in our cohort was 3.5%.

A nominal two-side *P*-value < 0.05 was considered to indicate statistical significance. All statistical analyses were performed using IBM SPSS Statistics version 27. Results from linear regressions are presented as B coefficients (β) with 95% confidence interval (CI), and results from logistic regressions are presented as odds ratios (OR) with 95% CI.

## Results

### Maternal characteristics

The study population consisted of 2498 women and child dyads with complete longitudinal data (after imputation of smoking status). The women were aged 16–45 years, 1037 (41.5%) were nulliparous, and 1010 (40.4%) were either overweight or obese (Table 1). The median gestational age at the time of fat depth measurements was 132 days (range 67 days, interquartile range 129–135 days).

**Table 1:** Descriptive characteristics of the study population.

	Variable	Cohort
Women	N	2498
	Age, years (mean, range)	30.3 (16–45)
	Nulliparous, n (%)	1037 (41.5)
	Early pregnancy BMI kg/m^2^ (mean ± SD)	25.1 ± 5.0
	Smoking at first antenatal visit, n (%)	88 (3.5)
	BMI < 18.5 kg/m^2^ (underweight), n (%)	60 (2.4)
	BMI 18.5–24.9 kg/m^2^ (normal weight), n (%)	1428 (57.2)
	BMI 25.0–29.9 kg/m^2^ (overweight), n (%)	621 (24.9)
	BMI ≥ 30.0 kg/m^2^ (obesity), n (%)	389 (15.6)
Offspring	N	2498
	Gestational length, days (mean ± SD)	279 ± 8
	Birthweight, g (mean ± SD)	3596 ± 479
	Small for gestational age, n (%)	250 (10.0)
	Large for gestational age, n (%)	250 (10.0)

*BMI* body mass index; small for gestational age, birthweight standard deviation score (BWSDS) below the 10th percentile in the study cohort; large for gestational age, BWSDS above the 90th percentile in the cohort.

### VF and SCF in relation to covariates

**Figure 1:**
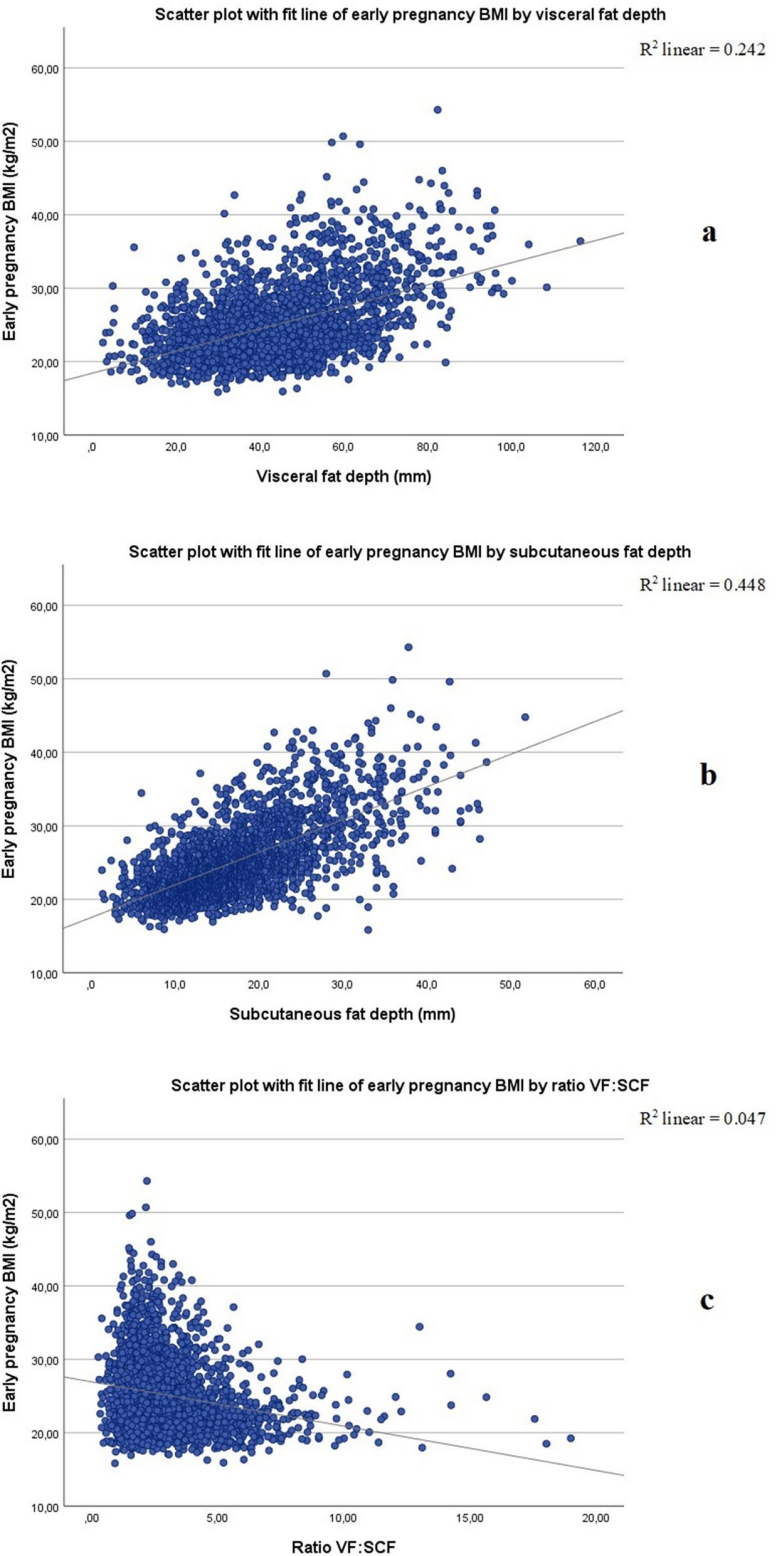
Scatter plots with fit line showing: (**a**) early pregnancy body mass index (BMI) by visceral fat depth (VF); (**b**) BMI by subcutaneous fat depth (SCF); and (**c**) BMI by ratio VF:SCF.

The overall range of VF was 3–116 mm and the range of SCF was 1–52 mm. The depth of both VF and SCF according to BMI class is presented in Supplementary Table S1. The depth of both VF and SCF (but not the ratio VF:SCF) increased with increasing BMI (Fig. 1), although the difference between BMI classes was not always significant (Supplementary Table S1). Of note, there was a wide variability in VF and SCF between individuals. Thus, there were cases of underweight women having greater VF and SCF than women with obesity class III (Supplementary Table S1).

Visceral fat depth and SCF were correlated with early pregnancy BMI (r = 0.49 and r = 0.67, respectively) (Supplementary Table S2). No correlations between maternal age and fat depths were found (Supplementary Table S2). Visceral fat depth was higher and SCF was lower in parous women compared with nulliparous women (Supplementary Table S3).

### Birthweight in relation to covariates

Parous women gave birth to heavier children compared with nulliparous women (Supplementary Table S4). Women born in EU gave birth to heavier children than did women born outside EU (Supplementary Table S4). The birthweight was lower among children whose mothers were smokers (Supplementary Table S4).

### Multivariable regression analyses on the association of VF and SCF with birthweight

For every 5-mm increase in VF there was an associated increase in birthweight of 19.5 g (95% CI 13.8–25.2 g), but this association was attenuated (8.3 g, 95% CI 2.5–14.1 g) after adjustment for covariates (Table 2). The unadjusted model showed that for every 5-mm increase in SCF, birthweight increased by 28.2 g (95% CI 15.9–40.6 g), but this association disappeared after adjustment for covariates (− 0.7 g, 95% CI − 15.4 to 13.9 g) (Table 2). The ratio VF:SCF was not associated with birthweight (Table 2). In the subgroup of healthy women, the results were similar (Supplementary Table S5). The impact of pre-pregnancy and pregnancy factors on infant birthweight is presented in Supplementary Table S6.

**Table 2:** Associations between maternal visceral fat depth (VF), subcutaneous fat depth (SCF), and the ratio VF:SCF on infant birthweight.

Outcome	Fat type	Unadjusted model			Adjusted model^a^		
		β	CI	*P*	β	CI	*P*
Birthweight (g)	VF	19.5	13.8 to 25.2	0.000	8.3	2.5 to 14.1	0.005
	SCF	28.2	15.9 to 40.6	0.000	− 0.7	− 15.4 to 13.9	0.921
	Ratio VF:SCF	5.8	− 4.7 to 16.3	0.277	7.2	− 2.4 to 16.8	0.139

Data are B coefficients (β) [95% confidence interval (CI)] for the change in outcome per 5 mm increase in fat depth and per unit increase in the ratio VF:SCF.

Data were analyzed using linear regression models.

^a^Adjustments in the model for VF: early pregnancy BMI, age, smoking at first antenatal visit, parity, SCF, country of birth, gestational age and infant sex. Adjustments in the model for SCF: early pregnancy BMI, age, smoking at first antenatal visit, parity, VF, country of birth, gestational age and infant sex. Adjustments in the model for ratio VF:SCF: early pregnancy BMI, age, smoking at first antenatal visit, parity, country of birth, gestational age and infant sex.

An increase in VF of 5 mm was associated with a 13% increase in the odds of having an infant born LGA (OR 1.13, 95% CI 1.09–1.17), but this association was attenuated after adjustment (OR 1.06, 95% CI 1.02–1.11) (Table 3). Overall, a 5-mm increase in SCF was associated with odds of LGA that were 16% higher (OR 1.16, 95% CI 1.07–1.26) (Table 3), but this association disappeared in the adjusted model (OR 0.95, 95% CI 0.85–1.07). The ratio VF:SCF was not associated with LGA in the unadjusted model, but after adjustments, an increase in VF:SCF by one unit was associated with odds of LGA that were 9% higher (OR 1.09, 95% CI 1.02–1.17) (Table 3). In the subgroup of healthy women, the results were similar, except that there was no association between the ratio VF:SCF and LGA (Supplementary Table S7). The impact of pre-pregnancy and pregnancy factors on the likelihood of giving birth to and LGA infant is presented in Supplementary Table S8.

**Table 3:** Associations between maternal visceral fat depth (VF), subcutaneous fat depth (SCF), and the ratio VF:SCF on the likelihood of giving birth to an infant large for gestational age (LGA).

Outcome	Fat type	Unadjusted model			Adjusted model^a^		
		OR	CI	*P*	OR	CI	*P*
LGA	VF	1.13	1.09−1.17	0.000	1.06	1.02−1.11	0.009
	SCF	1.16	1.07−1.26	0.000	0.95	0.85−1.07	0.412
	Ratio VF:SCF	1.05	0.99−1.13	0.130	1.09	1.02−1.17	0.015

Data are odds ratios (OR) [95% confidence interval (CI)] for the change in outcome per 5 mm increase in fat depth and per unit increase in the ratio VF:SCF.

Data were analyzed using logistic regression models.

^a^Adjustments in the model for VF: early pregnancy BMI, age, smoking at first antenatal visit, parity, SCF, and country of birth. Adjustments in the model for SCF: early pregnancy BMI, age, smoking at first antenatal visit, parity, VF, and country of birth. Adjustments in the model for ratio VF:SCF: early pregnancy BMI, age, smoking at first antenatal visit, parity, and country of birth.

## Discussion

This study showed that higher maternal VF measured by ultrasound in early mid pregnancy was associated with an increase in birthweight and the odds of giving birth to an LGA infant, independent of early pregnancy BMI. The mechanisms underpinning the association between maternal visceral adiposity and infant birthweight are uncertain.

Our findings are in agreement with a similar, but smaller, study by Jarvie et al.^32^, where abdominal subcutaneous and visceral adipose tissue thickness were measured by ultrasound at mean 12.4 weeks of gestation in 45 pregnant women. The study shows that first trimester visceral adipose tissue thickness, but not abdominal subcutaneous adipose tissue thickness or first trimester BMI, is associated with birthweight centile. Besides, the authors report an association between first trimester visceral adipose tissue thickness and fetal cord plasma triglyceride at delivery. Of note, fetal cord blood was available from only 23 of the pregnancies^32^. In non-pregnant individuals, fat accumulation in visceral fat tissue and other organs is proposed to occur when the adipocytes in the subcutaneous fat tissue are unable to store triglycerides^48^. Jarvie et al. hypothesize that in pregnant women with visceral fat accumulation, the fatty acids are transported to the fetus by the placenta, instead of being accumulated in ectopic organs, subsequently leading to higher birthweight^32^.

Visceral adiposity is associated with insulin resistance and dyslipidemia^49^, but whether this association is causal or not is unclear^50^. Migda et al. report higher first trimester fasting glucose levels in women delivering macrosomic and LGA infants, suggesting that maternal insulin resistance in early pregnancy is an underlying cause of excessive fetal growth^51^. Tchernof et al.^50^ proposes two possible mechanisms by which visceral adiposity could cause an altered metabolic state and insulin resistance. Firstly, adipocytes in visceral fat tissue are more hyperlipolytic than adipocytes in subcutaneous fat tissue^52^. Thus, a large amount of visceral fat increases the load of free fatty acids passing through the portal vein to the liver. This affects the hepatic metabolism, leading to increased production of apolipoprotein-B, lipoproteins and glucose, and decreased insulin degradation in the liver, ultimately resulting in higher systemic insulin levels and insulin resistance^48^. Secondly, excessive visceral fat tissue infiltrated with macrophages acts pro-inflammatory, generating an insulin resistant state^53^. It is hypothesized that maternal insulin resistance and increased plasma glucose levels triggers hyperinsulinemia in the fetus, which stimulates fetal growth^54^. Higher plasma glucose levels also results in more energy available for the fetus^51^. In our sensitivity analysis on a healthy subgroup, type 1 and 2 diabetes mellitus and gestational diabetes mellitus were three of the excluded diagnoses. The results from the sensitivity analysis also showed an independent association between central adiposity and infant birth size. Clearly, if insulin resistance is the mechanism behind the observed association between visceral adiposity and increased birthweight in our material, it is not detected by today’s thresholds used in screening for gestational diabetes mellitus in Sweden.

We found a positive association between maternal VF and the risk of giving birth to an LGA infant. To our knowledge, this has not previously been investigated. However, other proxies of central adiposity and LGA have been evaluated, with contradictory results. Higher WHR increases the odds of LGA according to two studies^14,18^. In contrast, another study reports no association between WHR ≥ 0.85 and LGA or WHR 0.80–0.84 and LGA, but an association between maternal BMI ≥ 30 kg/m^2^ and LGA^55^. Waist circumference (WC) predicts LGA in one study^13^, but not in another^56^. The different results could be due to heterogeneous study populations and differences in time of anthropometry measurement in relation to conception. Prior to gestational week 9, only little fat accretion is taking place, but between gestational week 9–22, fat accretion is rapid^57^. Moreover, the proxies of central adiposity in these studies are less precise in determining the amount of visceral fat tissue than ultrasound, since both WC and WHR include SCF^58^.

We did not find any association between SCF and birthweight or LGA after adjustments for covariates. Only VF was associated with birthweight and the likelihood of giving birth to an LGA infant. These findings are in agreement with the idea that SCF is less metabolically dysfunctional than VF, since visceral fat accumulation is associated with insulin resistance, hyperlipidemia, and low-grade inflammation^9,10^.

Several methods can be used to estimate body fat distribution, such as WC, WHR, ultrasound, bioelectrical impedance analysis, dual energy X-ray absorptiometry, computed tomography (CT), and magnetic resonance imaging (MRI)^58^. The two latter (CT and MRI) are considered the golden standard methods for visceral fat assessment^58^, but due to ionizing radiation, high cost and time requirement, the methods are inappropriate for use among pregnant women. Instead, anthropometric measurements (WC and WHR) and ultrasound are feasible during pregnancy. However, WC and WHR only give indirect measures of central adiposity^58^, and could also be biased by the growing uterus, especially in late pregnancy^58,59^. In contrast, ultrasound can distinguish visceral and subcutaneous fat compartments^36^, and hence obtain precise measures of these tissues. Moreover, evaluation of the validity and reproducibility of ultrasound assessment of intra-abdominal fat tissue shows a strong correlation between ultrasound and CT scan measurements (r = 0.81; *P* < 0.001)^60^.

The strength of this study was the large and representative population-based cohort, consisting of nearly 2500 pregnant women, which makes the results representative of the regional population. Of note, the population of Uppsala County is well educated and has a high socioeconomic status compared with the general Swedish population. The results might therefore not be representable for women in other socioeconomic settings. Even though only a subgroup of the women who underwent a second trimester anomaly scan during the study period underwent VF and SCF measurements, we do not believe that selection bias was present since it was a coincidence whether a midwife trained in fat depth measurement performed the scan or not. Yet another strength was that maternal central adiposity was determined by ultrasound measurements of VF and SCF, two compartments that cannot be distinguished by less precise methods such as WC or WHR.

A limitation to this study was that smoking status in early pregnancy was missing for the majority of the cohort and therefore imputed. Furthermore, we did not have any data on gestational weight gain in relation to central adiposity on the women included in our cohort. Gestational weight gain is associated with infant birthweight^61,62^, and could therefore have biased the results. Moreover, we did not have access to data on maternal height, which also could affect infant birth size. The fetal anomaly scans were performed during a wide time range regarding gestational age (range 67 days). However, the majority of the women had their scan performed in a narrower time interval, and there was no correlation between the timing of the measurements and fat depths. Hence, the timing of the measurements should not affect the overall results of this study.

## Conclusion

The current clinical practice to use maternal BMI to stratify the risk of adverse pregnancy and neonatal outcomes is insufficient, since BMI does not differentiate central from peripheral adiposity, or muscle mass from fat tissue. Therefore, a stronger predictor is needed to provide optimal care for overweight and obese pregnant women. We found that VF measured by ultrasound was associated with excessive fetal growth, independent of early pregnancy BMI, and may be useful in models for predicting LGA infants.

## Supplementary information

Supplementary Tables.

## Data Availability

The datasets generated during and/or analyzed during the current study are available from the corresponding author on reasonable request.

## References

[1] Santos S (2018). Gestational weight gain charts for different body mass index groups for women in Europe, North America, and Oceania. BMC Med..

[2] Hales CM, Fryar CD, Carroll MD, Freedman DS, Ogden CL (2018). Trends in obesity and severe obesity prevalence in US youth and adults by sex and age, 2007–2008 to 2015–2016. JAMA.

[3] Cnattingius S, Bergstrom R, Lipworth L, Kramer MS (1998). Prepregnancy weight and the risk of adverse pregnancy outcomes. N. Engl. J. Med..

[4] Sebire NJ (2001). Maternal obesity and pregnancy outcome: A study of 287,213 pregnancies in London. Int. J. Obes. Relat. Metab. Disord..

[5] Ruager-Martin R, Hyde MJ, Modi N (2010). Maternal obesity and infant outcomes. Early Hum. Dev..

[6] Blomberg M (2013). Maternal obesity, mode of delivery, and neonatal outcome. Obstet. Gynecol..

[7] Derraik JG, Ahlsson F, Diderholm B, Lundgren M (2015). Obesity rates in two generations of Swedish women entering pregnancy, and associated obesity risk among adult daughters. Sci. Rep..

[8] Catalano PM (2003). Obesity and pregnancy—The propagation of a viscous cycle?. J. Clin. Endocrinol. Metab..

[9] Hamdy O, Porramatikul S, Al-Ozairi E (2006). Metabolic obesity: The paradox between visceral and subcutaneous fat. Curr. Diabetes Rev..

[10] Fox CS (2007). Abdominal visceral and subcutaneous adipose tissue compartments: Association with metabolic risk factors in the Framingham Heart Study. Circulation.

[11] Alberti KG, Zimmet P, Shaw J (2005). The metabolic syndrome—A new worldwide definition. Lancet (London, England).

[12] Suresh A (2012). Comparison of maternal abdominal subcutaneous fat thickness and body mass index as markers for pregnancy outcomes: A stratified cohort study. Aust. N. Zeal. J. Obstet. Gynaecol..

[13] Gao X (2017). The mutual effect of pre-pregnancy body mass index, waist circumference and gestational weight gain on obesity-related adverse pregnancy outcomes: A birth cohort study. PLoS ONE.

[14] Salem W, Adler AI, Lee C, Smith GC (2012). Maternal waist to hip ratio is a risk factor for macrosomia. BJOG Int. J. Obstet. Gynaecol..

[15] Cisneiros RM (2013). Visceral adiposity in the first half of pregnancy predicts newborn weight among adolescent mothers. J. Obstet. Gynaecol. Can. JOGC.

[16] Li S (2013). Central adiposity and other anthropometric factors in relation to risk of macrosomia in an African American population. Obesity (Silver Spring, Md.).

[17] Brown JE (1996). Maternal waist-to-hip ratio as a predictor of newborn size: Results of the Diana Project. Epidemiology (Cambridge, Mass.).

[18] Bo S (2003). Obesity or diabetes: What is worse for the mother and for the baby?. Diabetes Metab..

[19] Jolly MC, Sebire NJ, Harris JP, Regan L, Robinson S (2003). Risk factors for macrosomia and its clinical consequences: A study of 350,311 pregnancies. Eur. J. Obstet. Gynecol. Reprod. Biol..

[20] Boyd ME, Usher RH, McLean FH (1983). Fetal macrosomia: Prediction, risks, proposed management. Obstet. Gynecol..

[21] Langer O, Berkus MD, Huff RW, Samueloff A (1991). Shoulder dystocia: Should the fetus weighing greater than or equal to 4000 grams be delivered by cesarean section?. Am. J. Obstet. Gynecol..

[22] Beta J (2019). Maternal and neonatal complications of fetal macrosomia: Systematic review and meta-analysis. Ultrasound Obstet. Gynecol..

[23] Groenendaal F, Elferink-Stinkens PM, Perinatal R (2006). Hypoglycaemia and seizures in large-for-gestational-age (LGA) full-term neonates. Acta Paediatr. (Oslo, Norway: 1992).

[24] Modanlou HD, Dorchester WL, Thorosian A, Freeman RK (1980). Macrosomia–maternal, fetal, and neonatal implications. Obstet. Gynecol..

[25] Ju H, Chadha Y, Donovan T, O'Rourke P (2009). Fetal macrosomia and pregnancy outcomes. Aust. N. Zeal. J. Obstet. Gynaecol..

[26] Ahlsson F, Gustafsson J, Tuvemo T, Lundgren M (2007). Females born large for gestational age have a doubled risk of giving birth to large for gestational age infants. Acta Paediatr. (Oslo, Norway: 1992).

[27] Innes K, Byers T, Schymura M (2000). Birth characteristics and subsequent risk for breast cancer in very young women. Am. J. Epidemiol..

[28] Johnsson IW, Haglund B, Ahlsson F, Gustafsson J (2015). A high birth weight is associated with increased risk of type 2 diabetes and obesity. Pediatr. Obes..

[29] Surkan PJ, Hsieh CC, Johansson AL, Dickman PW, Cnattingius S (2004). Reasons for increasing trends in large for gestational age births. Obstet. Gynecol..

[30] National Health Service (NHS). *Overweight and pregnant.* https://www.nhs.uk/conditions/pregnancy-and-baby/overweight-pregnant/. Accessed 3 Mar 2019 (2017).

[31] Kennedy NJ (2016). Maternal abdominal subcutaneous fat thickness as a predictor for adverse pregnancy outcome: A longitudinal cohort study. BJOG Int. J. Obstet. Gynaecol..

[32] Jarvie EM (2020). Maternal adipose tissue expansion, a missing link in the prediction of birth weight centile. J. Clin. Endocrinol. Metab..

[33] Lindberger E, Sundström Poromaa I, Ahlsson F (2020). Impact of maternal central adiposity on infant anthropometry and perinatal morbidity: A systematic review. Eur. J. Obstet. Gynecol. Reprod. Biol. X.

[34] Ludvigsson JF (2015). Ethical aspects of registry-based research in the Nordic countries. Clin. Epidemiol..

[35] Petersson K (2016). Prenatal diagnosis in Sweden 2011 to 2013-a register-based study. BMC Pregnancy Childbirth.

[36] Armellini F (1990). The contribution of sonography to the measurement of intra-abdominal fat. J. Clin. Ultrasound JCU.

[37] Koo TK, Li MY (2016). A guideline of selecting and reporting intraclass correlation coefficients for reliability research. J. Chiropr. Med..

[38] World Health Organization. *Body Mass Index—BMI.* https://www.euro.who.int/en/health-topics/disease-prevention/nutrition/a-healthy-lifestyle/body-mass-index-bmi. Accessed 3 Mar 2019 (2019).

[39] Niklasson A (1991). An update of the Swedish reference standards for weight, length and head circumference at birth for given gestational age (1977–1981). Acta Paediatr. Scand..

[40] Gaudet L, Ferraro ZM, Wen SW, Walker M (2014). Maternal obesity and occurrence of fetal macrosomia: A systematic review and meta-analysis. Biomed. Res. Int..

[41] Goisis A, Remes H, Barclay K, Martikainen P, Myrskyla M (2017). Advanced maternal age and the risk of low birth weight and preterm delivery: A within-family analysis using finnish population registers. Am. J. Epidemiol..

[42] Ko TJ (2014). Parental smoking during pregnancy and its association with low birth weight, small for gestational age, and preterm birth offspring: A birth cohort study. Pediatr. Neonatol..

[43] Hinkle SN (2014). The association between parity and birthweight in a longitudinal consecutive pregnancy cohort. Paediatr. Perinat. Epidemiol..

[44] Kiserud T (2018). The World Health Organization fetal growth charts: Concept, findings, interpretation, and application. Am. J. Obstet. Gynecol..

[45] Andridge RR, Little RJ (2010). A review of hot deck imputation for survey non-response. Int. Stat. Rev..

[46] Folkhälsomyndigheten. *Daglig tobaksrökning*. https://www.folkhalsomyndigheten.se/folkhalsorapportering-statistik/tolkad-rapportering/folkhalsans-utveckling/levnadsvanor/tobaksrokning-daglig/. Accessed 2 Mar 2020 (2019).

[47] Kreyberg I (2019). Stopping when knowing: Use of snus and nicotine during pregnancy in Scandinavia. ERJ Open Res..

[48] Despres JP (2008). Abdominal obesity and the metabolic syndrome: Contribution to global cardiometabolic risk. Arterioscler. Thromb. Vasc. Biol..

[49] Despres JP, Lemieux I (2006). Abdominal obesity and metabolic syndrome. Nature.

[50] Tchernof A, Despres JP (2013). Pathophysiology of human visceral obesity: An update. Physiol. Rev..

[51] Migda M, Migda MS, Migda B, Wender-Ozegowska E (2017). Maternal first trimester parameters in the prediction of excessive fetal growth in pregnant women with metabolic syndrome. J. Physiol. Pharmacol..

[52] Arner P (1995). Differences in lipolysis between human subcutaneous and omental adipose tissues. Ann. Med..

[53] Curat CA (2006). Macrophages in human visceral adipose tissue: Increased accumulation in obesity and a source of resistin and visfatin. Diabetologia.

[54] Ahlsson F (2010). Insulin resistance, a link between maternal overweight and fetal macrosomia in nondiabetic pregnancies. Hormone Res. Paediatr..

[55] McDonnold M (2016). Waist-to-hip ratio versus body mass index as predictor of obesity-related pregnancy outcomes. Am. J. Perinatol..

[56] Retnakaran R (2017). Maternal pre-gravid cardiometabolic health and infant birthweight: A prospective pre-conception cohort study. Nutr. Metab. Cardiovasc. Dis. NMCD.

[57] Butte NF, Ellis KJ, Wong WW, Hopkinson JM, Smith EO (2003). Composition of gestational weight gain impacts maternal fat retention and infant birth weight. Am. J. Obstet. Gynecol..

[58] Shuster A, Patlas M, Pinthus JH, Mourtzakis M (2012). The clinical importance of visceral adiposity: A critical review of methods for visceral adipose tissue analysis. Br. J. Radiol..

[59] McCarthy EA, Strauss BJ, Walker SP, Permezel M (2004). Determination of maternal body composition in pregnancy and its relevance to perinatal outcomes. Obstet. Gynecol. Surv..

[60] Stolk RP (2001). Validity and reproducibility of ultrasonography for the measurement of intra-abdominal adipose tissue. Int. J. Obes. Relat. Metab. Disord..

[61] Gaillard R (2013). Risk factors and outcomes of maternal obesity and excessive weight gain during pregnancy. Obesity (Silver Spring, Md.).

[62] Tian C (2016). Excessive weight gain during pregnancy and risk of macrosomia: A meta-analysis. Arch. Gynecol. Obstet..

