# Author Correction: Association of maternal central adiposity measured by ultrasound in early mid pregnancy with infant birth size

**DOI:** 10.1038/s41598-022-05233-8

**Published:** 2022-01-19

**Authors:** Emelie Lindberger, Anna-Karin Wikström, Eva Bergman, Karin Eurenius, Ajlana Mulic-Lutvica, Inger Sundström Poromaa, Fredrik Ahlsson

**Affiliations:** Department of Women’s and Children’s Health, Uppsala University, 751 85 Uppsala, Sweden

Correction to: *Scientific Reports* 10.1038/s41598-020-76741-8, published online 12 November 2020

The Supplementary Tables file published with this Article contained an error in Table S6. “Impact of pre-pregnancy and pregnancy factors on infant birthweight” where the β and CI values for the Variable 'Maternal country of birth outside EU' were incorrect. The correct and incorrect values appear below.

Incorrect:

**Table:** 

Outcome	Variable	β	CI	P
**Birthweight (g)**	Maternal country of birth outside EU	144.9	96.3–193.4	< 0.000

Correct:

**Table:** 

Outcome	Variable	β	CI	P
**Birthweight (g)**	Maternal country of birth outside EU	− 144.9	− 193.4 to − 96.3	< 0.000

This error has now been corrected in the Supplementary Tables file that accompanies the original Article.

## Supplementary Information

Supplementary Tables.

