# Supplementary material for: Association of maternal central adiposity measured by ultrasound in early mid pregnancy with infant birth size
Source: Sci Rep. 2020 Nov 12;10:19702. doi: 10.1038/s41598-020-76741-8 (PMC7665175; doi:10.1038/s41598-020-76741-8)
Supplement: Supplementary file 1 — Supplementary Tables. [file 41598_2020_76741_MOESM1_ESM.pdf]

Supplementary Information file

Association of maternal central adiposity measured by ultrasound in early mid pregnancy with infant birth size

Emelie Lindberger MD[1*], Anna-Karin Wikström MD PhD[1], Eva Bergman MD PhD[1], Karin Eurenius MD PhD[1], Ajlana Mulic-Lutvica MD PhD[1], Inger Sundström Poromaa MD PhD[1], Fredrik Ahlsson MD PhD[1]

[1]Department of Women's and Children's Health, Uppsala University, 751 85 Uppsala, Sweden

Table S1. Visceral and subcutaneous fat measures in relation to BMI classes defined by the World Health Organization.

BMI, WHO classification (kg/m^2)		Visceral fat (mm)		Subcutaneous fat (mm)	
	n	Mean ± SD	Range	Mean ± SD	Range
< 18.5 (Underweight)	60	33 ± 11[a]	11 – 61	10 ± 5[b]	3 – 33
18.5 – 24.9 (Normal weight)	1428	39 ± 13[b]	3 – 85	14 ± 5[b]	1 – 43
25.0 – 29.9 (Overweight)	621	46 ± 15[b]	5 – 98	19 ± 6[b]	2 – 46
30.0 – 34.9 (Obesity class I)	259	58 ± 16[c]	5 – 108	25 ± 7[c]	6 – 46
35.0 – 39.9 (Obesity class II)	98	63 ± 17[d]	10 – 116	29 ± 7[d]	13 – 47
≥ 40 (Obesity class III)	32	69 ± 17[e]	32 – 96	33 ± 7[e]	21 – 52

[a] significantly different from all other WHO BMI-classes, P <0.05. Kruskal-Wallis test followed by post-hoc paired tests with Bonferroni correction.

[b] significantly different from all other WHO BMI-classes, P <0.001. Kruskal-Wallis test followed by post-hoc paired tests with Bonferroni correction.

[c] significantly different from all other WHO BMI-classes, P <0.001, except obesity class II and III. Kruskal-Wallis test followed by post-hoc paired tests with Bonferroni correction.

[d] significantly different from all other WHO BMI-classes, P <0.001, except obesity class I and III. Kruskal-Wallis test followed by post-hoc paired tests with Bonferroni correction.

[e] significantly different from all other WHO BMI-classes, P <0.001, except obesity class I and II. Kruskal-Wallis test followed by post-hoc paired tests with Bonferroni correction.

BMI, body mass index.

Table S2. Pearson correlation coefficients for the linear associations between birth weight standard deviation score (BWSDS) and several clinical and demographic characteristics of the women.

	**Early pregnancy BMI**	**Age**	**Visceral fat**	**Subcutaneous fat**
**Offspring BWSDS**	0.21**	0.04*	0.14**	0.11**
**Early pregnancy BMI**		0.01	0.49**	0.67**
**Age**			0.08**	-0.02
**Visceral fat**				0.33**

BMI, body mass index.

*P < 0.05, **P < 0.01.

Table S3. Comparison of visceral fat and subcutaneous fat depth measures between groups defined by clinical and demographic parameters.

	Parity		Smoking at first antenatal visit		Woman's place of birth	
	**Nulliparous**	**Parous**	**Non-smoker**	**Smoker**	**EU**	**Outside EU**
**n**	1037	1461	2410	88	2164	334
**Visceral fat (mm)**	43 ± 16	45 ± 17*	44 ± 16	45 ± 17	44 ± 16	48 ± 16***
**Subcutaneous fat (mm)**	18 ± 7	16 ± 8***	17 ± 7	19 ± 9	17 ± 8	17 ± 7

EU, European Union.
Data are means ± standard deviations.
*$P < 0.05$ and ***$P < 0.001$ for a difference in outcome measure within a given parameter, from independent t-tests.

Table S4. Birthweight and birthweight standard deviation score (SDS) in associations with clinical and demographic parameters.

	Parity		Smoking at first antenatal visit		Woman's place of birth	
	**Nulliparous**	**Parous**	**Non-smoker**	**Smoker**	**EU**	**Outside EU**
**n**	1037	1461	2410	88	2164	334
**Birthweight (g)**	3500 ± 457	3663 ± 483***	3601 ± 478	3438 ± 497**	3616 ± 477	3464 ± 476***
**Birthweight SDS**	0.01 ± 0.93	0.46 ± 0.98***	0.29 ± 0.98	-0.03 ± 1.06**	0.32 ± 0.98	-0.00 ± 1.00***

EU, European Union.
Data are means ± standard deviations.
**P < 0.01 and ***P < 0.001 for a difference in outcome measure within a given parameter, from independent t-tests.

Table S5. Associations between maternal visceral fat depth (VF), subcutaneous fat depth (SCF), and the ratio VF:SCF on infant birthweight in a subgroup of healthy women (n = 2261).

Outcome	Fat type	Unadjusted model			Adjusted model [a]		
		β	CI	*P*	β	CI	*P*
**Birthweight (g)**	VF	21.0	15.0 − 27.0	0.000	9.8	3.7 − 15.8	0.002
	SCF	30.5	17.4 − 43.5	0.000	5.2	-10.0 to 20.4	0.504
	Ratio VF:SCF	5.4	-5.4 to 16.2	0.329	5.6	-4.3 to 15.4	0.268

Data are B coefficients (β) and (95% confidence interval (CI)) for the change in outcome per 5 mm increase in fat depth and per unit increase in the ratio VF:SCF.

[a] Data were analyzed using multivariable linear regression models. Adjustments in the model for VF: early pregnancy BMI, age, smoking at first antenatal visit, parity, SCF, country of birth, gestational age and infant sex. Adjustments in the model for SCF: early pregnancy BMI, age, smoking at first antenatal visit, parity, VF, country of birth, gestational age and infant sex. Adjustments in the model for ratio VF:SCF: early pregnancy BMI, age, smoking at first antenatal visit, parity, country of birth, gestational age and infant sex.

Table S6. Impact of pre-pregnancy and pregnancy factors on infant birthweight.

Outcome	Variable	β	CI	*P*
**Birthweight (g)**	Visceral fat depth (5 mm)	8.3	2.5 – 14.1	0.005
	Subcutaneous fat depth (5 mm)	-0.7	-15.4 to 13.9	0.921
	Early pregnancy BMI (kg/m^2)	15.3	10.5 − 20.2	<0.000
	Age (years)	-2.7	-6.3 to 0.8	0.134
	Smoker at first antenatal visit	-177.1	-266.5 to -87.7	<0.000
	Parous	199.4	163.6 – 235.3	<0.000
	Maternal country of birth outside EU	-144.9	-193.4 to -96.3	<0.000
	Gestational age (days)	23.4	21.4 – 25.4	<0.000
	Male infant	80.6	47.8 – 113.5	<0.000

Data are presented as the B coefficients (β) and (95% confidence interval (CI)) for the change in birthweight per unit change in the indicated variable after adjustments of all other listed variables. Data were analyzed using multivariable linear regression model.

Table S7. Associations between maternal visceral fat depth (VF), subcutaneous fat depth (SCF), and the ratio VF:SCF on the likelihood of giving birth to an infant large for gestational age (LGA) in a subgroup of healthy women (n = 2261).

**Outcome**	**Fat type**	**Unadjusted model**			**Adjusted model** [a]		
		OR	CI	*P*	OR	CI	*P*
**LGA**	VF	1.13	1.08 − 1.17	0.000	1.06	1.01 − 1.12	0.012
	SCF	1.18	1.09 − 1.29	0.000	1.00	0.90 − 1.13	0.952
	Ratio VF:SCF	1.02	0.94 − 1.10	0.659	1.05	0.97 − 1.13	0.214

Data are odds ratios (OR) (95% confidence interval (CI)) for the change in outcome per 5 mm increase in fat depth and per unit increase in the ratio VF:SCF.

Data were analyzed using logistic regression models.

[a] Adjustments in the model for VF: early pregnancy BMI, age, smoking at first antenatal visit, parity, SCF, and country of birth. Adjustments in the model for SCF: early pregnancy BMI, age, smoking at first antenatal visit, parity, VF, and country of birth. Adjustments in the model for ratio VF:SCF: early pregnancy BMI, age, smoking at first antenatal visit, parity, and country of birth.

Table S8. Impact of pre-pregnancy and pregnancy factors on the likelihood of giving birth to an infant large for gestational age (LGA).

**Outcome**	**Variable**	OR	CI	*P*
**LGA**	Visceral fat depth (5 mm)	1.06	1.02 − 1.11	0.009
	Subcutaneous fat depth (5 mm)	0.95	0.85 − 1.07	0.412
	Early pregnancy BMI (kg/m^2)	1.08	1.04 − 1.12	<0.000
	Age (years)	1.00	0.97 – 1.03	0.840
	Smoker at first antenatal visit	0.41	0.16 – 1.05	0.064
	Parous	2.40	1.73 – 3.33	<0.000
	Maternal country of birth outside EU	0.49	0.30 − 0.80	0.004

Data are presented as the odds ratios (OR) (95% confidence interval (CI)) for the change in outcome per unit change in the indicated variable after adjustments of all other listed variables. Data were analyzed using multivariable logistic regression model.
